# Spatial orientation, postural control and the vestibular system in healthy elderly and Alzheimer’s dementia

**DOI:** 10.7717/peerj.15040

**Published:** 2023-05-02

**Authors:** Mariya K. Chepisheva

**Affiliations:** Department of Brain Sciences, Division of Neurology, Faculty of Medicine, Imperial College London, London, United Kingdom

**Keywords:** Vestibular, Spatial orientation, Postural control, Falls, Healthy ageing, Alzheimer’s disease

## Abstract

**Background:**

While extensive research has been advancing our understanding of the spatial and postural decline in healthy elderly (HE) and Alzheimer’s disease (AD), much less is known about how the vestibular system contributes to the spatial and postural processing in these two populations. This is especially relevant during turning movements in the dark, such as while walking in our garden or at home at night, where the vestibular signal becomes central. As the prevention of falls and disorientation are of serious concern for the medical service, more vestibular-driven knowledge is necessary to decrease the burden for HE and AD patients with vestibular disabilities.

**Overview of the article:**

The review briefly presents the current “non-vestibular based” knowledge (*i.e*. knowledge based on research that does not mention the “vestibular system” as a contributor or does not investigate its effects) about spatial navigation and postural control during normal healthy ageing and AD pathology. Then, it concentrates on the critical sense of the vestibular system and explores the current expertise about the aspects of spatial orientation and postural control from a vestibular system point of view. The norm is set by first looking at how healthy elderly change with age with respect to their vestibular-guided navigation and balance, followed by the AD patients and the difficulties they experience in maintaining their balance or during navigation.

**Conclusion:**

Vestibular spatial and vestibular postural deficits present a considerable disadvantage and are felt not only on a physical but also on a psychological level by all those affected. Still, there is a clear need for more (central) vestibular-driven spatial and postural knowledge in healthy and pathological ageing, which can better facilitate our understanding of the aetiology of these dysfunctions. A possible change can start with the more frequent implementation of the “vestibular system examination/rehabilitation/therapy” in the clinic, which can then lead to an improvement of future prognostication and disease outcome for the patients.

## Introduction

Spatial orientation and postural control are essential for our autonomy as individuals. Proper navigation allows us to move freely in the environment and guarantees that daily needs can be met. Postural control, as a separate and, at the same time, highly linked aspect, ensures that navigation happens stably for the body (*i.e*. no falls), enabling proper exploration of the environment and successful reaching of our spatial goals.

During healthy ageing, spatial and postural processing accuracy decreases over time. This can sometimes lead to falls and/or disorientation and, in turn, to injuries, decreased self-esteem, and reduced independence through activities of daily living (ADLs). However, the changes observed in a prevalent neurological disorder of the central nervous system, such as Alzheimer’s disease, are even more profound. At presentation, a third of Alzheimer’s patients complain of getting lost during visually-guided orientation in their local environment (Henderson, Mack & Williams, 1989), and some might also report experiencing postural impairment, even early in the disease (Agrawal, Smith & Rosenberg, 2020; Yesantharao et al., 2022). Thus, even if postural imbalance is not a highly recognised feature of early Alzheimer’s disease, it becomes a serious concern later in time, with AD patients falling twice as much a year as compared to HE (Yesantharao et al., 2022). In trying to avoid the huge burden that imbalance and disorientation can have on affected individuals, enormous research effort is performed to explore how the spatial and postural modalities develop during healthy and pathological ageing. The literature presents a good number of highly informative reviews/opinions on the topic, and interested readers are invited to refer to Burgess, Maguire & O’Keefe (2002), Rubenstein (2006), Moffat (2009), Vann, Aggleton & Maguire (2009), Haddad et al. (2013), Vlček & Laczó (2014), Mesbah et al. (2016), Belghali et al. (2017), Lester et al. (2017), Coughlan et al. (2018), Cuevas-Trisan (2019), Diersch & Wolbers (2019), Segen et al. (2021).

Alongside vision and proprioception, the ***vestibular system*** is yet another sensory system crucial for our spatial navigation and postural control. However, its importance is often underestimated, and the vestibular system is seen as a complement to other sensory systems (please refer to Chepisheva (2021) for a detailed overview of the vestibular system and its spatial and postural aspects). Thus, while often taken for granted, the vestibular system remains “unnoticed” before it fails and starts to act abnormally (Guedry, 1974). It remains overlooked even in HE and AD patients, thus leading to a noticeable gap in the knowledge of two populations prominently affected by vestibular impairment (Harun et al., 2016; Agrawal et al., 2020).

In the dark, it is the vestibular system that helps us orientate during angular movements such as turns and rotations, *e.g*. while walking in our garden or at home at night, and contributes to our perception of self-motion and position in space (Seemungal et al., 2004; Grabherr et al., 2008; Cullen, 2019). Interestingly, brain research has not yet reported a link that mechanistically connects spatial orientation and postural control processing. However, a recent study in acute traumatic brain injury (TBI) patients (Calzolari et al., 2021) indicated that the right inferior longitudinal fasciculus (rILF) might represent a circuit where vestibular-based postural and spatial impairment meet. This finding suggests a neuronal correlate that might link vestibular spatial (*i.e*. *via* the vestibular self-motion perception) and vestibular postural processing. Even if this finding presents an interesting notion, it renders further affirmation through bigger sample-sized/longitudinally traced groups in different neurological conditions. Importantly, from a vestibular system point of view, vestibular self-motion perception and vestibular-guided spatial orientation might not be the same processes and could even be processed separately, as suggested in stroke patients with lesions in the TPJ (Kaski et al., 2016). Still, vestibular self-motion perception is a form of basic vestibular-guided “spatial orientation” (Agrawal, Smith & Rosenberg, 2020) and can naturally be referred to as such.

Thus, the simultaneous discussion of current scientific findings about vestibular spatial orientation and vestibular postural control in HE and AD patients in this review is based on the assumption that (1) spatial and postural processing might show a link *via* the central vestibular system. In addition, (2) postural control is a crucial part of spatial navigation, and (3) both processes mutually impact the quality of life of HE and Alzheimer’s patients as two faculties concurrently engaged in ecological behaviour such as foraging. By initiating this simultaneous discussion, the author hopes “to encourage further vestibular-based spatial and postural research and improve the outcome and disease management” (Chepisheva, 2021) for HE and AD patients affected by vestibular dysfunction. Importantly, discussion of all the spatial and postural literature in HE and AD or early biomarkers for pathological ageing through the modalities of orientation and balance are outside the scope of this review, and interested readers will be directed *via* citations mentioned throughout.

## Survey methodology

This literature review was performed in English and applies to articles available in English language only. Open and closed-access articles from PubMed and Google Scholar were reviewed.

Search words for spatial navigation in HE included: “spatial navigation + healthy elderly/healthy ageing/with age”, “spatial orientation + healthy elderly/healthy ageing/with age”, “spatial disorientation + healthy elderly/healthy ageing/with age”, “spatial deficits + healthy elderly/healthy ageing/with age”, ”spatial memory + healthy elderly/healthy ageing/with age”, “spatial + throughout life”, “visuospatial + healthy elderly/healthy ageing/with age”, “spatial + imaging + healthy elderly/healthy ageing”, “spatial + cognition + healthy elderly/healthy ageing”.

Further, search words for postural control in HE included: “postural control/postural stability/balance/falls + healthy elderly/healthy ageing/with age”, “postural deficits + healthy elderly/healthy ageing/with age”, “postural + throughout life”, “postural control/postural stability/balance/falls + imaging + healthy elderly/healthy ageing”, “postural control/postural stability/balance/falls + cognition + healthy elderly/healthy ageing”.

Next, “vestibular” was added to “healthy elderly/healthy ageing/with age”, and the “spatial” and “postural” searches were expanded with “vestibular/vestibular system/vestibular dysfunction/vestibular deficits”. Lastly, a combination of “spatial—postural—vestibular—HE” search words was performed.

The search strategy for Alzheimer’s patients was the same as for HE. The only difference consisted in replacing the “healthy elderly” with “Alzheimer’s disease/Alzheimer’s patients”/“Alzheimer’s”/“AD”/“Alzheimer’s dementia”/“getting lost + Alzheimer’s”/“falls + Alzheimer’s”.

This article discusses human-based research. Still, animal research is also mentioned as it often presents the only answers to current issues in human medicine. The literature search resulted in an extensive number of articles whose abstracts were reviewed. From them, 941 were downloaded and after a more detailed check, less than 200 articles remained to be included.

## Spatial navigation in healthy and pathological ageing

“***Spatial navigation***” is a multimodal process that requires dynamic updating of the relationship between the body and the environment (Berthoz & Viaud-Delmon, 1999), and “***spatial orientation***” as part of spatial navigation, is the ability to determine the position or direction of objects to their surrounding environment. This review does not aim to distinguish between spatial navigation and spatial orientation, and these terms have been interchangeably used.

There are two main strategies used during navigation: (1) ***egocentric***—referenced to the body or self, where information is derived from the vestibular, proprioceptive and visual (optic-flow) systems, and (2) ***allocentric***—concerning unchanging landmarks in the environment (O’Keefe & Nadel, 1978), where information is derived from external sources such as the visual, tactile and olfactory stimuli. Even if allocentric and egocentric spatial strategies are a crucial part of our navigation, they do not develop at the same time. Egocentrism is found across the whole life span, starting from infancy, and thus egocentric navigation is the first to be experienced. In contrast, allocentric skills are supposed to develop around the age of 7 years (Piaget & Inhelder, 1948). Later in adolescence, people tend to show a preference for the use of allocentric rather than egocentric strategies (Ruggiero, D’Errico & Iachini, 2016), possibly responding to the more complex environment they interact with. Even so, research suggests that both types of strategies are equally established and available to use, as indicated by the use of the sequential egocentric and allocentric strategies (Iglói et al., 2009). However, with increasing age, healthy elderly start to show a tendency towards an egocentric navigational strategy (Moffat, 2009; Harris & Wolbers, 2013; Lester et al., 2017), similar to pathological individuals, such as Alzheimer’s or mild cognitive impairment (MCI) patients. This way, the process of strategy selection throughout the lifespan resembles an inverted U curve (Ruggiero, D’Errico & Iachini, 2016), with childhood and old age relying primarily on egocentric skills and adolescents and adults—on allocentric.

### Healthy ageing—non-vestibular spatial research

Spatial navigation studies in human healthy ageing have shown a progressive loss of spatial navigational abilities with increasing age (Wilkniss et al., 1997; Moffat, Zonderman & Resnick, 2001, Moffat, 2009; Lester et al., 2017; Fernandez-Baizan et al., 2019), alongside a decline in episodic memory (Hedden & Gabrieli, 2004) and spatial working memory (Reuter-Lorenz et al., 2000; Holden & Gilbert, 2012). Age-related differences have been observed in brain regions crucial for spatial navigation, such as the hippocampus, parahippocampal gyrus and retrosplenial cortex and, more generally—the medial parietal lobe. Accompanying posterior to anterior neuronal activity shift and increased activation in the anterior cingulate gyrus and medial frontal lobe were also observed (Moffat, Elkins & Resnick, 2006, Moffat, 2009; Park, 2010; Diersch et al., 2019), possibly as a compensatory mechanism for visually-guided spatial tasks.

Studying the spatial navigational decline in the elderly means that deficits in multiple spatial abilities must be reviewed. Some examples suggest (1) decreased spatial updating in a virtual maze task that was learned passively while seated (Zancada-Menendez et al., 2015), (2) less efficient pattern separation in spatial memory, which is essential for the enhancement of memory accuracy within the hippocampus (with the dentate gyrus and CA3 hippocampal region implicated by ageing) (Holden & Gilbert, 2012), and (3) decreased performance on the route re-tracing but not on route repetition task that can be solved with an egocentric strategy (Wiener, Kmecova & de Condappa, 2012). Further examples include (4) decreased cerebral oxygenation in the dorsolateral prefrontal cortex that was associated with decreased visual-spatial performance (Kronovsek et al., 2021), (5) less varied spatial and psychomotor strategies in spatial place learning tasks (Davis & Weisbeck, 2015), and (6) decreased speed of processing and episodic memory due to age-related decline in dopamine receptors (Bäckman et al., 2000), which are essential for spatial navigation.

However, spatial performance in the elderly does not show only a decline but often also preservation of spatial skills please check Park (2010), Rosenbaum et al. (2012), Konishi & Bohbot (2013). Indeed, a study reports that if HE were given a chance to actively navigate during training instead of passively encoding while seated in a chair in front of a computer screen, they showed a significant improvement when re-tracing the learned virtual route (Meade et al., 2019). Even if the authors do not discuss the vestibular system as a possible contributor to this improvement of results, it is worth mentioning that the actual exploration of the environment contributed to the generation of multiple sensory cues, among which also vestibular cues, which, in turn, might have helped re-trace the virtual route. Still, research also shows that even when we are seated in front of a computer, our brain’s compensatory mechanisms *via* the optic-flow can ensure a speed signal *via* the triggering of activity in the vestibular nuclei even if the vestibular system itself is inactivated (Jacob et al., 2014). Thus, it is expected that some vestibular signals would be available also in passive virtual reality testing, even if not as strong as during a real-world exploration.

Consequently, even if navigation has been recognised as an aspect of cognitive function that is at risk during healthy ageing (Moffat, 2009), the reasons for the spatial decline are not purely cognitive, as various research has shown its complex nature relying on multiple modalities. Numerous compensatory mechanisms are in place to keep normally ageing individuals able of successful navigation, and the discovery of spatial navigational cells such as place cells, head direction (HD) cells and grid cells “adds a unique assessment feature to the cognitive ageing toolbox” (Moffat, 2009).

### Healthy ageing—vestibular spatial research

The Baltimore Longitudinal Study of Ageing (USA) has significantly contributed to our understanding of the peripheral vestibular loss and spatial impairment in healthy elderly. Still, the interpretation of vestibular loss is complex, and more so the discrimination between a central and a peripheral cause (Brandt, Strupp & Dieterich, 2014). As different aspects need to be considered, researchers point out that “often the deterioration in vestibular functioning relates to how the signal is processed by the brain circuits rather than an impairment in the sensory transduction process” itself (Arshad & Seemungal, 2016).

Interestingly, a study discussing an active navigational task in healthy ageing [mean age + SD = 69.8 years ± 13.6] suggested a statistically notable link between a decrease in the saccular function (*i.e*. otoliths) and an impaired angular and distance perception in the Triangle Completion test (Anson et al., 2019a). This is not an expected result, given that the otolith impairment is not directly related to angular perception, especially when the authors reported no link between semicircular canal function and heading errors. Even if (1) “saccular stimulation leads to widespread cortical activation, including the posterior insular cortex, the inferior parietal cortex, the intraparietal sulcus, and temporo-parietal junction”, as discussed in Harun et al. (2016) and even if (2) vertical movements/gravitational cues are indeed a crucial part of our spatial navigation (also in the horizontal plane), the result of the study is unclear. Thus, the implication of the saccular function alone in this “complex outcome” warrants further investigation, and the authors were cautious of overinterpretation by suggesting that this link was not causal and might have been due to some visuospatial cognitive impairment and a possible reduction of multisensory perception (Anson et al., 2019a).

Neuroimaging studies from the same research group also stressed the link between saccular impairment and hippocampal volume loss in HE (Kamil et al., 2018). A detailed investigation of the volume and shape of vestibular-related regions showed a reduced volume of the hippocampus and the entorhinal cortex corresponding (among other brain regions) to a decreased saccular function in the elderly (Jacob et al., 2020). Even if these results prove to be of interest, further studies are needed to establish such a central role of the saccule in human (mostly horizontal) navigation, and especially during older age, when little climbing (limited primarily to climbing the stairs) or any extreme vertical (up-down) activities are performed. It is also important to note, that the otoconia in the saccule are suggested to deteriorate quicker than the otoconia in the utricle (Igarashi et al., 1993).

Further, a relationship between vestibular loss and (1) decreased topographical memory (Previc et al., 2014) and (2) decreased visuospatial abilities were reported (Bigelow et al., 2015), but the authors pointed out that additional evidence is needed to establish a causal role (Bigelow et al., 2015).

Interestingly, in another recent study, healthy elderly (mean age = 77 years) were examined for their peripheral vestibular function and invited to participate in a spatial task with a rotation chair. The results demonstrated that HE with a low vestibulo-ocular reflex (VOR) or bilaterally absent utricular function were less accurate than those with intact vestibular peripheries (Anson et al., 2021). Considering the often-seen difference between the results of the video Head Impulse Test (vHIT) and the caloric reflex test (*i.e*. calorics test), researchers suggested that either the bilateral horizontal semicircular canal or bilateral utricle impairment might contribute to the spatial angular disorientation (Anson et al., 2021). This study is one of only a few available examples where results could warrant a data-based discussion about the central vestibular processing in HE. However, as presented, the experimental design for the spatial rotation test indicates several aspects that cause unclarity. The main discrepancy arises as during the training phase, the participants experienced the exact same angular rotations and timings in the light that were later tested in the dark. In addition, the presentation of the allocentric cues on the walls of the laboratory in a clockwise fashion might have significantly contributed to the estimation of travelled distance with the help of the visual cues, while the task aimed to test “vestibular perceived travelled distance”. This, in turn, might have led to participants trying to remember the velocity of the outbound stimulus (later in the dark) and to calculate their position based on the learned association between “allocentric cues—velocity—timing of rotation” rather than relying predominantly on vestibular perception. The bias from this learning effect would become even more significant if a specific angle was associated only with one specific time duration. Eventually, this might turn into a test of a velocity and/or time-related spatial memory and thus miss its vestibular focus.

Generally, research in HE indicates that the cervical vestibular evoked myogenic potentials, *i.e*. cVemps (testing for saccular integrity) remain stable up to the age of 50–60 years, and the ocular vestibular evoked myogenic potentials, *i.e*. oVemps (testing for utricle integrity)-up to 60–80 years (Ji & Zhai, 2018). Thus, based on the studies reviewed so far, the testing of the saccular function might suggest being a candidate for a biomarker and a possible indicator of the need for timely rehabilitation so as to prevent further degeneration. Still, such an assumption needs to be taken with great caution as older adults’ cVemps (especially >60 years) might not always be present (Piker et al., 2013), thus limiting the ability of the cVemps to reveal a potential vestibular dysfunction. At this point, a more comprehensive testing strategy of both the semicircular canals and the otoliths would secure better results.

### Alzheimer’s disease—non-vestibular spatial research

Alzheimer’s disease (AD) is the most common form of dementia, with approximately 47 million people and increasing worldwide prevalence up to 76 million by 2030 (Mehta & Yeo, 2017; Alzheimer’s Association, 2021; Alzheimer’s Disease International, 2021). The disease results in a marked decline in navigation skills that are initially prominent in unfamiliar environments and later occur also in familiar settings.

Considering how multifaceted spatial navigation is, it is to be expected that spatial impairment in Alzheimer’s manifests as a decline in multiple navigational abilities (Vlček & Laczó, 2014). Some examples, among many, include (1) impaired ‘egocentric’ to ‘allocentric’ translation *via* the retrosplenial cortex (Cushman, Stein & Duffy, 2008; Morganti, Stefanini & Riva, 2013), (2) a decrease in optic-flow perception and general visuospatial abilities (Tetewsky & Duffy, 1999; O’Brien, 2001), sometimes more pronounced during dynamic visual activities than static visual activities (Rizzo & Nawrot, 1998), and consequent (3) impairment in mental rotation tasks (Adduri, Marotta & Giurfa, 2009), (4) disrupted scene matching (Lee et al., 2006), and (5) decreased spatial planning and problem-solving (Passini et al., 1995). However, there is still a limited understanding of the neural mechanisms underlying this decline (Pai & Jacobs, 2004; Stangl et al., 2018, Segen et al., 2021).

In fact, many people with Alzheimer’s disease are transferred to assisted care homes, and they are, in due course, confronted with having to learn a completely new environment within which to navigate (O’Malley, Innes & Wiener, 2017). Aimless navigation or wandering is yet a typical behaviour for AD patients, but as of now, there is no standardisation in the objective quantifying of wandering behaviour, with some feasibility studies being underway (Kamil et al., 2021). Thus, the need to better understand this progressive loss of navigational skills becomes even more pressing. Research has shown that people with dementia need spaces specifically designed for them to orientate successfully. Suggestions include (1) bright colours, (2) easily readable signs, (3) uniqueness in common rooms, (4) fewer decision points, (5) fewer objects in the room, and (6) also short corridors, (7) calm surroundings, and (8) more benches along the walking path to take a rest in case of a panic episode or disorientation (Passini et al., 2000; Landmark et al., 2009; Marquardt, 2011; O’Malley, Innes & Wiener, 2017; Cogné et al., 2018). For instance, when AD patients were assisted indoors with remotely controlled sound and light (Caffò et al., 2014) or outdoors *via* a specifically developed mobile device (Lanza et al., 2014), patients were able to orientate well. This type of support can help boost their confidence in making them feel more independent and consequently can serve as a stimulation to stay physically active. Still, longitudinal data and multi-cohort studies would greatly assist in the correct evaluation of (1) how helpful such procedures are in the long-term and whether patients continue to be active/feel involved, (2) what level of activity patients need so as to feel “stimulated” and not “intimidated”, and (3) how this affects their general activities of daily living (ADLs). Notably, such interventions should find their way not only into research-based centres but also into real care homes.

#### ‘Spatial navigation’ as a “Better” Biomarker for AD diagnosis

Although the decline in episodic memory has been a gold standard in diagnosing AD for a long time (Dubois et al., 2014), the pronounced spatial disorientation turns out to be a more sensitive biomarker than episodic memory (Johnson et al., 2009), as the brain regions affected early in the disease pathology are “key nodes in the spatial navigation network” (Coughlan et al., 2018). Thus, spatial navigation suggests being a better way to distinguish between AD patients and other individuals with memory loss. One such group are the healthy elderly who do not usually experience topographical disorientation or at least not in such a severe form as AD patients (Cushman, Stein & Duffy, 2008; Mokrisova et al., 2016; Coughlan et al., 2018). Further, spatial orientation tasks *via* a route learning procedure managed also to discriminate between Alzheimer’s and semantic dementia individuals (Pengas et al., 2010). Moreover, spatial orientation studies seem to similarly help in differentiating between AD and mild cognitive impairment (MCI) (Laczó et al., 2010; Moodley et al., 2015; Parizkova et al., 2018; Howett et al., 2019), also between subtypes of MCI (Laczó et al., 2011) and similarly among APOE ɛ sub-types and thus, even years before clinical onset (Bierbrauer et al., 2019; Coughlan et al., 2020).

With a wide variety of studies reporting a severe navigational decline in Alzheimer’s disease, it has remained ambiguous for a long time whether egocentric or allocentric navigational skills are the first to deteriorate during the disease. Early research in AD compared a real-world hidden goal task and a computer version of the same task to establish similar results between methodologies and significant allocentric impairment in the AD group compared to controls (Kalová et al., 2005). Further, research in amnestic MCI (aMCI) and AD patients showed that during a virtual Y-maze task for assessing strategy of navigation, patients expressed a higher preference for egocentric skills over allocentric corresponding to the severity of their condition: aMCI showed 67% preference for egocentric *vs* 33% for allocentric, AD showed 94% preference for egocentric *vs* 6%, while age- and education level matched healthy elderly—39% *vs* 61% (Parizkova et al., 2018). Moreover, both real-world and computer-based Morris Water Maze (MWM) established spatial impairment in aMCI, mild and moderate AD that was proportional to their right hippocampus (Nedelska et al., 2012; Parizkova et al., 2018) and basal forebrain volume loss (Parizkova et al., 2018).

Another important study supporting the idea of “allocentric first” decline is the PREVENT Dementia Program (Ritchie et al., 2018). It suggests that when examining the allocentric (with the Four Mountain task (Bird et al., 2010; Chan et al., 2016; Wood et al., 2016)) and egocentric (with the supermarket trolley task (Tu et al., 2015)) strategies in APOE ɛ4 carriers, then the allocentric deficit served as a better predictor of risk in the allele carriers than egocentric navigation or episodic memory performance. Thus, the earliest cognitive changes might be partially steered by tau-related pathology in the medial temporal lobe and not exclusively by amyloid depositions in the medial parietal lobe (Ritchie et al., 2018).

Lastly, as previously mentioned, spatial orientation serves as a biomarker not only for patients who are already experiencing cognitive decline but also for those who are referred to as risk groups, *i.e*. APOE ɛ carriers. Research shows that (1) within a large cohort study of 442 non-demented adults, the Floor Maze test successfully predicted pre-dementia syndromes based on immediate and delayed time to complete the test (Verghese, Lipton & Ayers, 2017). Further, (2) the hippocampus-based allocentric Four Mountain task suggested a high specificity as a biomarker for pre-clinical Alzheimer’s disease (Chan et al., 2016; Wood et al., 2016). However, (3) an entorhinal cortex (EC) based virtual reality path integration task discriminated better among patients positive *vs* negative for CSF amyloid-β and total tau pathology than standardised tests such as the ACE, Trail making or even the hippocampus-based Four Mountain task (Howett et al., 2019). Similarly, when using an EC-based hypothesis, genetic risk AD participants showed that right EC and posterior cingulate cortex (PCC) connectivity was lower in the ε3ε4 (high-risk) group and correlated with spatial navigation differences between the high-risk ε3ε4 and the low-risk ε3ε3 carriers (Coughlan et al., 2020).

### Alzheimer’s disease—vestibular spatial research

As discussed in detail in (Chepisheva, 2021), research has shown that vestibular dysfunction is associated with hippocampal damage (Smith, 1997; Brandt et al., 2005). However, no causal link between vestibular loss and cognitive decline has been established (Harun et al., 2016). Still, vestibular impairment is suggested to affect many domains of cognitive control, among which are spatial navigation, gait and locomotion (Camicioli et al., 1997; Stackman & Taube, 1997; Brandt et al., 2005; Smith, Darlington & Zheng, 2010; Smith & Zheng, 2013; Previc, 2013; Hitier, Besnard & Smith, 2014; Yoder & Taube, 2014; Seemungal, 2014; Mast et al., 2014; Previc et al., 2014; Bigelow & Agrawal, 2015; Smith, 2017, 2019, 2022). A detailed literature review reveals that while non-vestibular AD research on spatial navigation is highly abundant, peripheral vestibular spatial testing is less frequent but exists, whereas vestibular-guided spatial testing looking for central aetiology in Alzheimer’s pathology is rarely attempted at all.

In 2013, Prof. Previc discussed a “vestibular hypothesis” and made a strong case suggesting that peripheral vestibular loss needs to be more critically reviewed as it might be linked to the neurodegenerative decline in Alzheimer’s disease (Previc, 2013). In short, he discussed that the wide-spread vestibular network is strongly represented in the medial temporal lobe and that multiple risk factors for Alzheimer’s disease (*e.g*. age, traumatic brain injury and diabetes, among others) are also accompanied by vestibular impairment. In addition, multiple cholinergic fibres link the hippocampus and the vestibular system (Wei et al., 2018). A subsequent commentary to Prof. Previc’s article by Prof. Smith (Smith, 2013) gave a clear explanation that a patient diagnosed with AD might experience spatial navigational deficits and memory decline but still show a preserved VOR as the vestibular pathways contributing are different. Subsequent studies have indeed revealed no association between VOR and cognitive impairment (Harun et al., 2016). One possible mechanism of compensation that AD patients might use to stabilise their gaze and show a normal VOR gain is by reducing the optokinetic reflex gain (OKR) (Chong et al., 1999).

In a very recent study (Coughlan et al., 2022), “at genetic risk” AD participants (ε3ε4 carriers) and ε3ε3 carrier controls (ε classification based on saliva samples) were tested in a type of a vestibular-based spatial task (*i.e*. path integration) on a rotatory chair while in complete darkness. The discrepancies in the participants’ vestibular-derived performance were enough for an automated algorithm to differentiate between them with a 65–79% accuracy. Thus, the authors hoped that early identification of vestibular dysfunction might give better chances for “at genetic risk” individuals to receive targeted treatment. Even if an interesting candidate study for “central vestibular system” research, the study leaves an unclear message about the quality of its vestibular results. Briefly, the researchers did not report for peripheral or central vestibular testing to have taken place for the cohort aged 50–75 years. Consequently, it became unclear if the nature of the reported vestibular disadvantage for the ε3ε4 carriers was of peripheral or central aetiology, which would lead to different conclusions and intervention procedures. The testing procedure warrants further detail regarding the vestibular stimulus itself—the range and duration of angular rotations, the consistency in the way the vestibular stimulus was produced (in this case, manually by the researcher standing behind the rotating chair), peak velocity, the possibility of vestibular memory decay/velocity storage (von Brevern et al., 1997; Seemungal et al., 2004; Bronstein et al., 2008; Yoder & Taube, 2009), the presence of the researcher behind the chair and its effect on vestibular processing (Lopez et al., 2015), among others.

Another vestibular (peripheral vestibular) study reported a significant link between bilateral saccular dysfunction and self-reported driving difficulties in a visuospatial questionnaire in MCI and AD patients (Wei et al., 2017). Although this link was mediated by the spatial performance on the Money Road Map test, the data do not seem enough to imply a causal link. However, saccular impairment can positively be one of the multiple contributors to spatial navigation decline during everyday activities for neurodegenerative patients, similar to what research showed about healthy elderly.

In another research effort, scientists examined saccular, utricular and semicircular canal function in MCI and AD patients and observed more expressed impairment in the AD patients (but not in the MCI) in the otoliths (*i.e*. saccule and utricle) and not in the semicircular canals when compared to healthy elderly (Harun et al., 2016). Thus, AD patients showed more significant peripheral vestibular impairment (based on their otolith function only) than MCI (Harun et al., 2016).

Lastly, a more recent study (Wei et al., 2018) suggested once more that saccular vestibular loss may contribute to spatial deterioration in AD patients. However, this time it was irrespective of whether a general cognitive decline was already present or not, thus stressing that otolith-cervical and otolith-cortical projections are distinct (Harun et al., 2016). Following that research has shown that AD patients with vestibular loss (most probably authors meant “peripheral” vestibular loss) have “disproportionate levels of spatial cognitive impairment relative to AD patients without vestibular loss” (Agrawal, Smith & Rosenberg, 2020), it was suggested that vestibular dysfunction might lead to a “spatial” subtype of AD (Wei et al., 2018; Agrawal, Smith & Rosenberg, 2020). Still, considering that measuring the saccular function *via* the cVemps is an indirect measure of otolith activity (Ji & Zhai, 2018), the results in this and previous studies must be interpreted with caution.

## Postural control in healthy and pathological ageing

When moving in our environment, many of the issues we face are connected not only to the processing of information about the space around us, *i.e*. spatial information but also about balancing our posture, *i.e*. postural control. Both balance and postural control are an integral part of spatial navigation. “***Balance***” is the ability to retain a state of equilibrium without falling, and “***postural control***” is defined as “the act of maintaining, achieving or restoring a state of balance during any posture or activity” (Pollock et al., 2000). This review does not aim to distinguish between balance and postural control, and these terms have been interchangeably used.

A comprehensive review of the balance literature showed that balance, similar to spatial navigation is a “whole brain phenomenon” (Surgent et al., 2019) implicating multiple brain structures: the cerebellum, the basal ganglia, the thalamus, the hippocampus but also cortical regions such as the frontal cortex, the inferior parietal cortex and the putative “vestibular cortex”. Postural control itself, even when only “standing quietly”, is already an extremely complicated task and “depends on a remarkably complex sensorimotor control system” (Peterka, 2002). However, it is characterised by even more excessive and uncontrolled sway with increasing age and cognitive decline, especially in individuals with neurodegenerative diseases, such as Alzheimer’s disease (Haddad et al., 2013; Costa et al., 2016). Thus, quantifying gait, balance and postural control provide essential information contributing to our understanding of healthy ageing and neurological conditions affecting the central nervous system where “motor symptoms predominate and cause considerable functional impairment” (Buckley et al., 2019).

### Healthy ageing—non-vestibular balance research

Postural control and balance deteriorate with increasing age, putting HE at increased risk of falls, abnormal gait, injuries, institutionalisation, or fatal outcome (Tinetti, Speechley & Ginter, 1988; Rubenstein, 2006; Verghese et al., 2006; Agrawal et al., 2020). In particular, falls can lead to soft tissue injuries, longstanding pain, fractures and worse quality of life (Karlsson et al., 2013). Falls are often associated with unsteady gait, confusion, anxiety (Bloem, Steijns & Smits-Engelsman, 2003), general or muscle weakness or even with side effects of medications (Rubenstein, 2006). Thus, it is crucial to make a difference between internal (*e.g*. cognitive impairment, age, vision, musculoskeletal diseases, chronic arthritis, gait and balance disorders, side effects of medications) and external (*e.g*. immobile lifestyle, malnutrition, uneven or slippery floors, poor lighting, improper footwear) causes of falls as some of them might be modifiable, if properly looked into (Karlsson et al., 2013; Cuevas-Trisan, 2019). Indeed, research has shown that proper detection, more structured clinical examination procedures and timely management of those risk factors can reduce the chance of falls in the elderly (Rubenstein, 2006; Karlsson et al., 2013; Cuevas-Trisan, 2019).

Identifying the risk factors between fallers and non-fallers has been an important part of our understanding of how balance in HE can be preserved and falls can potentially be prevented. Research shows that the spontaneous lateral sway in a light-deprived condition (*i.e*. where vestibular and/or proprioceptive systems start to play a more central role) for HE aged 62–96 years is “the single best predictor of future falling risk” (Maki, Holliday & Topper, 1994). This idea is supported by further research reporting that vestibular noise in the roll-tilt condition in HE (age range 18–80 years) is associated with an inability to complete the “eyes-closed on a foam” condition (*i.e*. the vestibular condition) during the Romberg test (Karmali et al., 2017). This increased roll-tilt noise, in consequence, “should cause increased mediolateral sway” (Wagner, Chaudhari & Merfeld, 2021).

As an integral part of postural control and balance, the vestibular system is difficult to omit in the investigation of balance dysfunction, even if not directly addressed by many researchers. Thus, the author of the current review will refer to more articles about balance in HE in the section discussing vestibular-based postural research. Here, the author briefly looks into the competition between the postural and cognitive resources in HE through the prism of “dual tasking”. Indeed, “dual tasking” has proved to be a more useful task design than single postural tasks for the detection of the subtle abnormalities that arise during the postural/cognitive competition. For instance, a dual-task study investigated postural performance by measuring the centre of pressure (CoP) in young adults, elderly without a history of falls and elderly with a history of falls (Shumway-Cook et al., 1997). Generally, there was either no significant difference in postural stability on a firm surface between the young adults and elderly without a history of falls or it was a marginal one (Shumway-Cook et al., 1997). It was with the implementation of an additional task or an unstable surface (*e.g*. foam) that age-related differences started occurring (Shumway-Cook et al., 1997). Importantly, those with a history of falls showed impaired performance, irrespective of surface firmness, with performance worsening even more when a cognitive task was introduced (Shumway-Cook et al., 1997).

In another CoP study, researchers report that adding a cognitive task improved static postural stability in young adults (*i.e*. a “cognition first” strategy) but led to no change in older adults (*i.e*. a “posture first” principle) (Berger & Bernard-Demanze, 2011). Further, they also discuss specific strategies that the elderly perform to compensate for possible deterioration in balance, such as (1) more explorative movements to gain more proprioceptive information and thus maintain stability (Berger & Bernard-Demanze, 2011) or (2) increased use of a “hip strategy” as to increase the medial-lateral (ML) axis sway (Maki, Holliday & Topper, 1994; Berger & Bernard-Demanze, 2011).

The last two studies are examples of that a clear hierarchy of attentional resources has been shown to exist with ageing (Shumway-Cook et al., 1997). However, the authors argue that this does not mean that posture is more important hierarchically than any secondary (*i.e*. cognitive) task during dual tasking, as the observed decrements were in postural stability rather than cognitive performance (Shumway-Cook et al., 1997). Notably, the allocation of attention to balance or cognition depends greatly on the current situation (Shumway-Cook et al., 1997), *i.e*. is there any apparent danger to postural stability or not.

Even if a significant amount of consideration has been given to the early identification of the risk of falls in the healthy elderly, to really improve their postural stability and increase their independence, HE must be more regularly attracted to balance training programs. For this to happen, such programs have to implement some “fun” and/or engaging elements, such as the perturbation-based balance training which has been used to reduce the incidence of falls, both in healthy and pathological individuals (Gerards et al., 2017).

### Healthy ageing—vestibular balance research

Age-related decline in the sensory systems and a decreased ability to adapt to changes in the environment are typical for ageing (Osoba et al., 2019). Thus, the elderly tend to experience greater difficulties adjusting their balance, especially after sudden changes, with their reliance on visual cues increasing with age (Osoba et al., 2019). Healthy elderly women (mean age 73 ± 9 years) with observed normal somatosensory function showed an increased antero-posterior (AP) sway (during eyes-closed condition) that was significantly correlated to their MoCA performance (Leandri et al., 2015). Thus, the authors implicated the vestibular system as a possible link between balance and cognitive performance (Leandri et al., 2015). Still, in this study, the somatosensory function was measured based on “observation”, and no quantitative measure *via* ankle proprioceptors or plantar pressure sensors was used. Nevertheless, it was reported that the participants were regular gym visitors and had “no impairment of daily activity, or orthopedic, otolaryngologic, neurologic, or cardiovascular conditions.” (Leandri et al., 2015).

Further, a galvanic vestibular stimulation (GVS) study showed that in comparison to younger adults, HE could not find a proper motor strategy to compensate for the effect of the GVS. Consequently, this “negatively interfered with their ability to re-evaluate sensory information” (Carvalho et al., 2019) on time and confirmed once more the general decline in the semicircular canal and otolith function that occurs with age (Agrawal et al., 2012; Iwasaki & Yamasoba, 2014; Arshad & Seemungal, 2016; Ji & Zhai, 2018).

Another US-based study reported that more than 35% of people aged 40 years and over, accounting for 69 million Americans, have impaired balance due to peripheral vestibular impairment (Agrawal et al., 2009). This, in turn, has a significant influence on mortality and morbidity rates (Agrawal et al., 2009), as vestibular loss is known to contribute not only to postural instability but also to vertigo and dizziness (Fernández, Breinbauer & Delano, 2015), which might alone or in combination with other factors lead to falls (Agrawal et al., 2009; Liston et al., 2014). Indeed, around 35–40% of community-dwelling HE fall every year (Rubenstein, 2006; Etman et al., 2012), many of them more than once, and around 70%—acquire an injury after the fall (Stel et al., 2004; Talbot et al., 2005). An examination of a group of community-dwelling healthy elderly (70–95 years) showed that half of them had an abnormal HIT, which was associated with reduced gait speed and an increased number of reported falls during the last year (Agrawal et al., 2013). Interestingly, further investigation of the spatial and temporal aspects of gait revealed that longer and slower steps were associated with horizontal semicircular canal impairment but not with otolith dysfunction (Anson et al., 2019b). Please refer to Horak, Shupert & Mirka (1989), Yim-Chiplis & Talbot (2000), Baloh, Ying & Jacobson (2003), Horak (2006), Sturnieks, George & Lord (2008), Peterka (2018), Osoba et al. (2019), Agrawal et al. (2020), Coto et al. (2021), Wagner et al. (2021), Wagner, Chaudhari & Merfeld (2021) for further reviews on the balance dysfunction and the vestibular system in HE.

A neuroimaging study showed that age-related white matter disruption (through fractional anisotropy (FA) investigation) in the bilateral occipital forceps was the best predictor of imbalance in the vestibular condition of the sensory organization test (SOT) (van Impe et al., 2011). Another DTI study investigated age-related decline in the white matter (WM) of the vestibular spinal tract (VST) and the parieto-insular vestibular cortex (PIVC) (Yeo, Kwon & Cho, 2020). Researchers found that a decreased WM tract volume in the medial and lateral VST and PIVC and decreased mean FA in the lateral VST in the elderly might be associated with postural impairment (Yeo, Kwon & Cho, 2020).

However, clinicians state that not enough is being done for vestibular damage in the elderly to be accurately diagnosed and timely managed (Zalewski, 2015). Screening for vestibular impairments and targeted vestibular therapy may be an essential (Agrawal et al., 2013) part of a change that needs to happen in the healthcare system for HE to be adequately supported. If really implemented, vestibular therapies can be helpful—*e.g*. an Internet-based vestibular rehabilitation trial to reduce dizziness due to vestibular dysfunction was attempted in the elderly (median age 67 years). It showed a reduction of dizziness and dizziness-related disabilities in the patients who continued to perform the exercises and demonstrated its potential to help a vast majority of affected people in the community without the need for an onsite visit (Geraghty et al., 2017). Therefore, specialised training can improve balance, and thus even up to the age of 99 years (Deems, Deems & O’Malley, 2019). Consequently, the implementation of vestibular therapy can realistically lead to a reduction of falls leaving older healthy and possibly even pathological individuals more independent while safely performing multiple activities common to their daily life.

### Alzheimer’s disease—non-vestibular balance research

People with Alzheimer’s disease are at a high risk of falls, and even if falls are a less recognised feature of AD, these patients are two to three times more likely to fall and get an injury as compared to healthy elderly (Montero-Odasso et al., 2012; Cronin, Arshad & Seemungal, 2017; Agrawal, Smith & Rosenberg, 2020). This may be connected to the fact that falls often come before cognitive decline and thus may remain “unnoticed”.

Having recognised the profound consequences that falls might have for AD patients, scientists and clinicians have addressed this gap by conducting an immense amount of postural-based research *via* multiple testing techniques (*e.g*. 3D motion capture, force plates, instrumented mats, inertial measurement units (IMU), accelerometers and gyroscopes) and in diverse testing conditions (*e.g*. quiet single or double-leg stance, tandem or Romberg stance, firm or unstable surface with eyes-open or eyes-closed, with backward or forward inclination, sit to stand task, turning 360°, picking up an object from the floor) (Mesbah et al., 2016; Buckley et al., 2019). Even if non-vestibular postural studies (*i.e*. those that are not “discussing” the vestibular system or its contribution to posture) are not the main scope of this review, the author briefly mentions such studies, among which also some discussing gait. The latter is done to show that “gait is emerging as a potential diagnostic tool for cognitive decline” (Mc Ardle et al., 2018), also in a dual-task design (Montero-Odasso et al., 2012).

For instance, a custom-based IMU wearable device has shown its capacity to automatically analyse gait and balance patterns in AD patients in different situations without any optical or camera sensors, as traditionally done (Hsu et al., 2014). This turns such an IMU into an easy-to-use tool that can collect a significant amount of data and possibly help to develop an early diagnostic device based on postural measurements (Hsu et al., 2014). In addition, the body-worn sensor devices can inform about gait performance at home and in the clinic as they are well tolerated by participants (Mc Ardle et al., 2018). Notably, while collecting data for a specific individual, they have the potential to establish a personalised training plan concerning this patient’s specific needs across time. However, further effort is required to standardise those various approaches and increase their sensitivity to specific neurological conditions, both when identifying subtle postural and gait abnormalities in the prodromal state and later during disease progression (Buckley et al., 2019).

A study revealed that the most common reason for falling in AD was stumbling, with those with lower MMSE scores experiencing more falls (Perttila et al., 2017). Reasonably, AD patients who were more physically active, received less medication and ate regularly experienced fewer falls than their counterparts with more medications and generally worsened medical conditions, sometimes accompanied by a further medical diagnosis, *e.g*. COPD, osteoarthritis, diabetes mellitus (Perttila et al., 2017).

When looking at a standard task design, such as “dual-tasking”, a comprehensive review (Belghali et al., 2017) revealed some patterns of dual-task related gait changes in Alzheimer’s disease, mentioning among others changes in pace (*e.g*. speed, stride length, swing and stance time variability), rhythm and postural control (*e.g*. asymmetry in stride width and length). One of the first studies to establish a link between mental load and abnormalities during locomotion (*i.e*. during dual-tasking) reported that “talking while walking” leads to a significant decline of speed in Alzheimer’s patients performing a verbal fluency task (*i.e*. reciting names) while walking (Camicioli et al., 1997). The posture first principle discussed in the “non-vestibular balance research in HE” can well apply here. Using cognitive resources to compensate for postural control inevitably leads to the inability to address the “anticipating” postural changes during walking, which are a form of a cognitive modality. Thus, at first glance, pure postural preference might be enough to ensure stable posture for AD patients, but further consideration reveals that such a strategy is not rigid in the long-term postural safety of the individual, as “adaptation”/“anticipation” is a crucial part of balance. The reason is that posture is not a purely mechanistic task (Manckoundia, Pfitzenmeyer & Athis, 2006) but a complex, cognitively involved process.

Further, scientists reported an increased CoP area and path for AD patients compared to HE, with these differences increasing in the dual-task condition (Manckoundia, Pfitzenmeyer & Athis, 2006). Thus, the authors discuss that (1) AD patients have an impaired ability to coordinate activities and that (2) standing still requires attention and is not a purely “overlearned” mechanistic task (Manckoundia, Pfitzenmeyer & Athis, 2006). Other examples of the effect of attention on postural performance include tasks with auditory (Wittwer, Webster & Hill, 2013), visual (Jor’dan et al., 2015), or auditory and visual (Gago et al., 2015) distractions. The latter study (Gago et al., 2015) discussed a generally increased postural sway while background noise was available and immediate improvement when it was removed. Still, compared to visual suppression, auditory cues had less effect on the postural decline.

Dual-tasking was even used as a biomarker for the prediction of MCI to dementia progression—out of 112 participants, 27 progressed to dementia, and 23 of them—to AD dementia (Montero-Odasso et al., 2017). While slow single-task velocity was not a predictive measure, gait abnormalities during counting backwards and listing animal names were indicative of future dementia progression (Montero-Odasso et al., 2017). Thus, the authors argue that although further data are needed to support these findings, the straightforward design of dual-task testing makes it a proper addition to the clinical examination procedure to help guide the planning and management of MCI patients (Montero-Odasso et al., 2012, 2017; Bahureksa et al., 2017). Importantly, other studies stress the fact that arithmetic (*e.g*. such as counting backward) rather than pure semantic (*e.g*. such as listing animal names) tasks are better at identifying the subtle changes (Bahureksa et al., 2017).

Consequently, any putative (vestibular) rehabilitation should focus on rather challenging motor and cognitively demanding tasks that can improve the cognition-posture relationship (Bahureksa et al., 2017). However, controversy exists on whether participation of AD patients in balance training programs can show any benefits for the patients, with some reporting positive results (De Andrade et al., 2013; Debove et al., 2017), while others—no effect (Toots et al., 2016). Unfortunately, the 2021 report of Alzheimer’s Disease International titled “World Alzheimer Report 2021, Journey through the diagnosis of dementia” confirms that almost 33% of the interviewed clinicians (*n* = 3,542) believe that after a dementia diagnosis, nothing can be done or changed (Alzheimer’s Disease International, 2021). This, in turn, might have negative consequences as the success of such balance programs relies not only on the fact whether it is specialised and targeted to the specific patients, but also on the engagement of the medical staff.

### Alzheimer’s disease—vestibular balance research

Strong evidence exists that static and functional postural stability is impaired in Alzheimer’s disease as compared to healthy elderly (Mesbah et al., 2016), and peripheral vestibular loss might contribute to this decline (Biju et al., 2022). What is more, attentional demand in AD during dual-task activity and loss of visual input (*i.e*. when vestibular and/or proprioceptive systems are left active) were shown to be key factors contributing to postural instability in Alzheimer’s patients (Mesbah et al., 2016).

Increased antero-posterior sway was observed in aMCI and AD patients during an eyes-closed posturography condition, thus implying a possible vestibular and/or somatosensory impairment (Leandri et al., 2009). Moreover, the AP sway showed that balance is an issue not only for AD but also for aMCI, in addition to proving to be a sensitive measure to discriminate between AD patients and ‘aMCI and controls’ (Leandri et al., 2009). Importantly, the patients in the latter study were screened for extrapyramidal signs and had no history of “recorded falls, or relevant cardiovascular, metabolic, rheumatologic, orthopedic or other neurological conditions.” (Leandri et al., 2009).

Furthermore, the reduced ability to suppress sensory stimuli (as seen in the Alzheimer’s disease non-vestibular balance research section) and improper use of attentional resources led scientists to examine various postural conditions with a mixture of congruent and incongruent visual and somatosensory cues. A study showed that AD patients (tested for an intact vestibular system *via* VOR and optokinetic reflexes (OKR)) were unable to retain proper postural control under ‘vestibular system demanding conditions’ while suppressing incongruent visual and somatosensory information, which sometimes led to falls (Chong et al., 1999). However, they showed stable posture when only the somatosensory input was incongruent (Chong et al., 1999). Interestingly, AD patients also showed a relatively small sway on the eyes-closed condition compared to eyes-open, thus clearly indicating they are not as dependent on vision as the healthy controls (Chong et al., 1999). This way, the authors suggested that suppression of the visual input might have decreased the sensory demand on the Alzheimer’s brain (Chong et al., 1999) instead of being seen as a “loss of visual information”.

A virtual reality-based study (Gago et al., 2016) performed unexpected visual displacements to examine the provoked compensatory postural adjustments in AD patients with the help of kinematic and time-frequency analyses on IMUs (Gago et al., 2016). Although these patients were reported to have an intact vestibular system, the authors do not discuss how they examined it. *Via* the virtual reality, the environment challenged the visual, proprioceptive and vestibular cues (*e.g*. by including illusional self-motion), contributing to a slowed reweighting of sensory input and increased compensatory postural disturbance in the patients (Gago et al., 2016). The authors conclude that insufficient cognitive resources, hippocampal atrophy and existing fear of falling might be essential contributors to the general inability to rapidly re-adjust after unexpected body perturbations (Gago et al., 2016).

Interestingly, considering the strong correlation between the orientation component of the Alzheimer’s Disease Assessment Scale (ADAS) and the balance performance in the eyes-closed condition in AD patients, Leandri et al. (2009) suggested that “balance impairment could be related to reduced hippocampal performance” (Leandri et al., 2009). Making this statement, Leandri et al. cited Brandt et al. (2005), even if the latter article does not make a direct statement about such a link in bilateral vestibular failure patients. The unclear link between hippocampal atrophy and postural imbalance appears again in Gago et al. (2016). In particular, “some studies have reported that the hippocampus uses vestibular information for spatial memory and navigation, and that balance impairment could be related to reduced hippocampal performance” (Gago et al., 2016). Gago et al. made this claim based on their read of the Smith (1997) article, even if the latter article (Smith, 1997) does not make a direct statement. Several other attempts have been made to find a link between static/dynamic postural control and the hippocampal volume in MCI and/or healthy elderly patients, still with no real association established (Beauchet et al., 2015, 2016). Importantly, correlation is not causation, and a link between hippocampal atrophy and postural imbalance is at this point (1) only correlational and not causative, even in the studies where some link/association was established and (2) for such a link to be implied, much further investigation is needed.

Further, researchers observed that during caloric vestibular stimulation the visual suppression rate of AD patients prone to falls was lower than in those who were not as imbalanced (Nakamagoe et al., 2015). Thus, since peripheral vestibular system function was found to be preserved, researchers assumed a possible central vestibular system abnormality, making it one of the very few examples in the literature where the central vestibular system in Alzheimer’s patients is discussed based on experimental postural control evidence. Additionally, the AD faller group single photon emission computed tomography (SPECT) images revealed a decreased blood flow in the inferior parietal lobule (IPL), which is an essential part of the putative “vestibular cortex”. This led to a suggested association between visual suppression, vestibular imbalance and damage in the IPL (Nakamagoe et al., 2015).

Interestingly, in unilateral vestibular hypofunction (UVH) patients with MCI, AD or normal cognitive level, researchers reported an increased sway in both eyes-closed and eyes-open condition for the static posturography test for AD patients, but not for MCI and pure UVH patients (Micarelli et al., 2018). Importantly, ipsilesional VOR gain was reduced but similar (*i.e*. between 0.61–0.63) among the three groups, confirming that it is not linked to cognitive decline. Consequently, it was suggested that “central signal integration” (Micarelli et al., 2018) is processed differently among the three cognitive groups with UVH.

Another recent study Biju et al. (2022) reported that, similar to Chong et al. (1999), AD patients with preserved semicircular canal and otolith function had better balance performance during the eyes-closed condition (the study did not control for proprioception). However, AD patients showing some peripheral vestibular impairment (for both, semicircular canal and otoliths) experienced more medial-lateral sway (both in eyes-open and eyes-closed conditions). Thus, they showed results in concordance with other vestibular disorders (Biju et al., 2022) and some healthy elderly (Maki, Holliday & Topper, 1994). Authors explain their results by mentioning that ML sway is more biomechanically challenging than AP and is therefore expected to degrade quicker (Biju et al., 2022). This result stands in contrast with Leandri et al. (2009) mentioned previously, where AP sway was more pronounced. Leandri et al. (2009) discussed that “a fault in the balance controlling mechanisms of the central nervous system would more readily affect the anteroposterior balance” (Leandri et al., 2009), whereas “latero-lateral stability is more due to anatomical than functional mechanisms, because the involved joints have very little range of motion in this plane” (Leandri et al., 2009).

Finally, although postural instability and falls present a great risk for the AD population, scientists reveal that out of 801 AD patients at a tertiary AD referral centre, only 48 were sent to standard physical therapy and only five of them (*i.e*. 0.62%)—to vestibular physical therapy (Gandhi, Klatt & Agrawal, 2020). Even if not all AD patients will eventually benefit from such treatment, a change in the medical practice is warranted as it will help at least some AD patients and improve their physical and psychological health (Agrawal, Smith & Rosenberg, 2020; Yesantharao et al., 2022). A new clinical trial that is testing the benefit of vestibular therapy on balance and spatial cognitive skills in AD patients has recently started (Yesantharao et al., 2022). This trial plans to recruit 100 AD patients and follow them up to 1 year post-therapy.

## Conclusion

A well-established decline in the peripheral vestibular system (Agrawal, Smith & Rosenberg, 2020) is observed with increasing age, which might affect not only postural control and falls but also spatial aspects such as topographical memory, visuospatial abilities and navigation. Even if the vestibular system is only one of many contributors to spatial and postural processing, the loss of vestibular function in the elderly has been shown to significantly decrease their quality of life and incur “a substantial societal cost” (Agrawal, Pineault & Semenov, 2017). While for healthy individuals, the vestibular system presents as a crucial resource ensuring precise and coordinated navigation and postural control, it is unclear if the diseased brain evaluates this extra sensory information as (1) an additional chance for a compensatory mechanism or rather as (2) a “noisy” burden to the already “computationally” overloaded pathological brain. Thus, even if healthy elderly present with a certain decrease in these functions, the amount to which they have deteriorated in AD patients is much greater, limiting AD patients’ abilities to compensate for any abnormalities present.

Multiple invasive/non-invasive high-level neurotechnological approaches have been taken both towards the diagnosis and treatment of AD pathology (Ning et al., 2022). Among these are combinations of multi-omic, multi-neuroimaging, computational brain-network modelling (Yu, Sporns & Saykin, 2021), multi-targeted pharmaceutical and other non-pharmacological approaches (Hou et al., 2019). Unfortunately, decades of research steered initially towards the amyloid ß (Makin, 2018; Panza et al., 2019) and later including the tau-pathology hypotheses (Mullard, 2021) have yet not been successful in revealing the complete aetiology of Alzheimer’s disease. Considering its complex nature and the genetic and environmental factors affecting the disease progression, “single-drug or single-pathway-targeted approaches” have shown to be inadequate (Hou et al., 2019). On the other hand, combined treatment strategies and multifaceted approaches show more promise (Elmaleh et al., 2019; Hou et al., 2019; Yu, Sporns & Saykin, 2021; Whitson et al., 2022).

Whereas the knowledge about how the vestibular loss contributes to spatial and postural processing is unlikely to solve the ambiguity surrounding healthy and pathological ageing, the vestibular system and its importance in the overall solution of this issue should not be underestimated. As already stated, a more holistic approach, coming from all possible contributors, should weigh the importance of all systems involved. Thus, the goal of this review was to concentrate on one of the often underrated but crucial contributors to the general solution of this ambiguity, *i.e*. the vestibular system. As seen throughout this review, even if there is some human-based expertise about the peripheral vestibular system and its link to spatial and postural impairment, the studies attempting central vestibular research are very few. Even so, these few central vestibular studies presented some unclarities about their results, making it impossible for us to gain further knowledge about central vestibular dysfunction in the navigation/balance of healthy elderly and AD patients. Consequently, this review (1) emphasises the need for more vestibular-based spatial and postural research in HE and AD patients and (2) stresses the gaps in knowledge that exist around this research.

Given the immense effect disorientation and balance loss/falls can play in one’s life, alongside the many physical, mental and financial implications connected with them, researchers suggested that “given that vestibular impairment may represent a modifiable risk factor for cognitive decline and AD, the impact of vestibular loss on cognition should be considered a critical new area of investigation” (Agrawal, Smith & Rosenberg, 2020). One possible way the medical practice might take to better address the high percentage of vestibular dysfunction, both in HE and Alzheimer’s individuals, is (1) the implementation of more vestibular-based examinations and vestibular-based rehabilitation therapies. However, such a change can only happen with the help of more feasibility studies and solid scientific evidence stressing the crucial role that the vestibular system can have for the long-term solution and understanding of the neural mechanisms of the spatial and postural modalities in HE and AD patients.

Another path for the medical service involves (2) the “vestibular implants”. Vestibular implants have been successfully developed not only for animals (Karmali et al., 2021) but also for humans (Chow et al., 2021). In the human-based study, patients improved their balance and gait, but all of them experienced some hearing loss. This was an expected side effect leading to the vestibular implant being placed only unilaterally instead of bilaterally (Chow et al., 2021). Thus, many questions about the nature of the vestibular system and its involvement in spatial orientation and postural control have been answered but even more—remain open. Consequently, much work is ahead aiming at better facilitation of neurological diagnoses and improved treatment schemes (Montero-Odasso et al., 2017) for patients with vestibular disabilities.

## Additional Information and Declarations

### Data Availability

The following information was supplied regarding data availability: This is a literature review.
